# The 1.7 Å X-Ray Crystal Structure of the Porcine Factor VIII C2 Domain and Binding Analysis to Anti-Human C2 Domain Antibodies and Phospholipid Surfaces

**DOI:** 10.1371/journal.pone.0122447

**Published:** 2015-03-16

**Authors:** Caileen M. Brison, Steven M. Mullen, Michelle E. Wuerth, Kira Podolsky, Matthew Cook, Jacob A. Herman, Justin D. Walter, Shannon L. Meeks, P. Clint Spiegel

**Affiliations:** 1 Western Washington University, Department of Chemistry, Bellingham, Washington, 98225-9150, United States of America; 2 Aflac Cancer and Blood Disorders Center, Department of Pediatrics, Emory University, Atlanta, Georgia, 30322, United States of America; University of Pennsylvania School of Medicine, UNITED STATES

## Abstract

The factor VIII C2 domain is essential for binding to activated platelet surfaces as well as the cofactor activity of factor VIII in blood coagulation. Inhibitory antibodies against the C2 domain commonly develop following factor VIII replacement therapy for hemophilia A patients, or they may spontaneously arise in cases of acquired hemophilia. Porcine factor VIII is an effective therapeutic for hemophilia patients with inhibitor due to its low cross-reactivity; however, the molecular basis for this behavior is poorly understood. In this study, the X-ray crystal structure of the porcine factor VIII C2 domain was determined, and superposition of the human and porcine C2 domains demonstrates that most surface-exposed differences cluster on the face harboring the “non-classical” antibody epitopes. Furthermore, antibody-binding results illustrate that the “classical” 3E6 antibody can bind both the human and porcine C2 domains, although the inhibitory titer to human factor VIII is 41 Bethesda Units (BU)/mg IgG versus 0.8 BU/mg IgG to porcine factor VIII, while the non-classical G99 antibody does not bind to the porcine C2 domain nor inhibit porcine factor VIII activity. Further structural analysis of differences between the electrostatic surface potentials suggest that the C2 domain binds to the negatively charged phospholipid surfaces of activated platelets primarily through the 3E6 epitope region. In contrast, the G99 face, which contains residue 2227, should be distal to the membrane surface. Phospholipid binding assays indicate that both porcine and human factor VIII C2 domains bind with comparable affinities, and the human K2227A and K2227E mutants bind to phospholipid surfaces with similar affinities as well. Lastly, the G99 IgG bound to PS-immobilized factor VIII C2 domain with an apparent dissociation constant of 15.5 nM, whereas 3E6 antibody binding to PS-bound C2 domain was not observed.

## Introduction

Hemophilia A is an X-linked bleeding disorder resulting from dysfunctional blood coagulation factor VIII (fVIII), affecting 1 in 5,000 males worldwide. The most effective treatment for hemophilia A consists of repeated therapeutic infusions of either plasma-derived or recombinant fVIII, commonly referred to as fVIII replacement therapy [1–3]. There are significant clinical complications to replacement therapy, whereby approximately 30% of patients receiving therapy develop inhibitory antibodies against fVIII, thus rendering the replacement therapy ineffective [4–6]. By contrast, spontaneous fVIII inhibitory antibodies may also develop against endogenous, functional fVIII in other populations, resulting in acquired hemophilia [7]. Factor VIII inhibitory antibody development consists of partial or complete inhibition of the cofactor function of fVIII, resulting in loss of proper hemostasis.

Blood coagulation fVIII is expressed as a 2,332-residue glycoprotein cofactor, yielding the domain architecture: A1-A2-B-*ap*-A3-C1-C2 [8, 9]. The three A domains form a trimeric structure homologous to the copper binding protein, ceruloplasmin [10]. The C1 and C2 domains project from the trimeric A domain assembly, are homologous to the discoidin family of protein folds, and are essential for negatively-charged phospholipid membrane binding as well as cofactor activity [11–15]. In circulation, fVIII is bound to von Willebrand factor (VWF) multimers as a single chain or an inactive heterodimer [16–19]. Upon proteolytic activation by either thrombin or factor Xa (fXa), activated fVIII (fVIIIa) becomes a heterotrimeric assembly (A1/A2/A3-C1-C2), which dissociates from VWF and binds to activated platelet surfaces with the serine protease, factor IXa (fIXa), to form the intrinsic “tenase” complex, which efficiently converts fX to fXa [20–23]. The presence of fVIIIa enhances the activity of fIXa for the proteolytic activation of fX by approximately 200,000-fold [20, 21].

**Fig 1:**
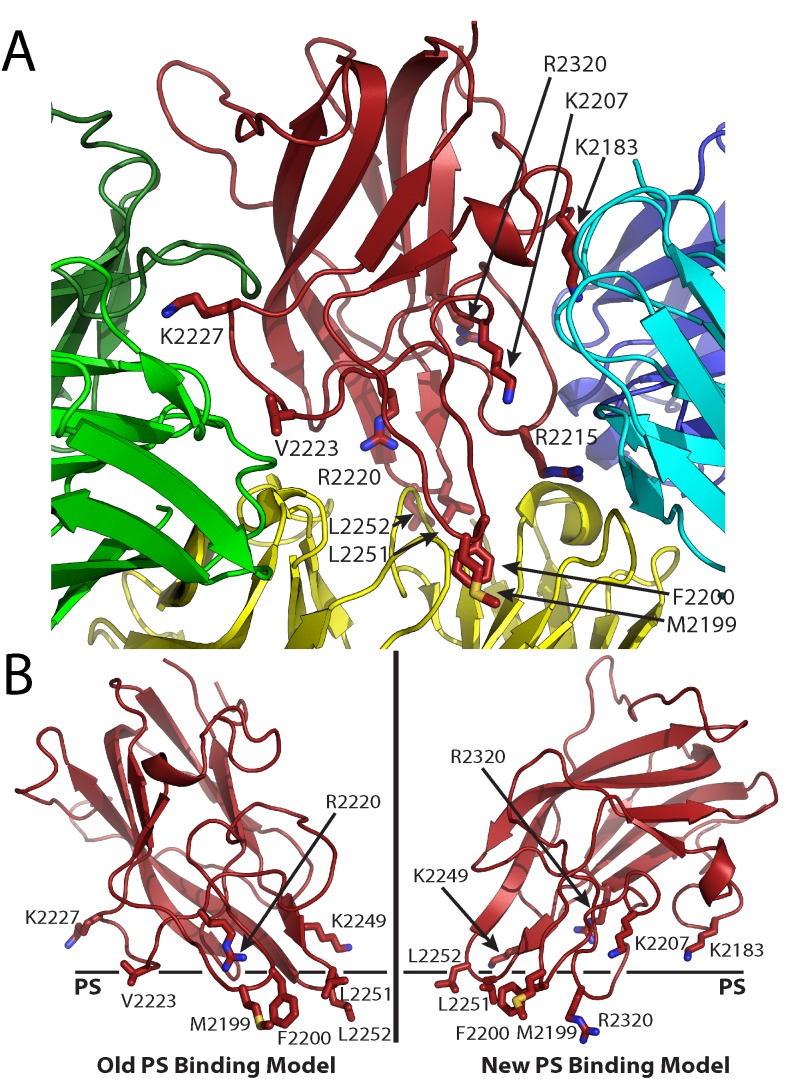
Factor VIII C2 domain-specific inhibitory antibody epitopes and membrane binding models. (A) Ribbon diagram representation of the fVIII C2 domain bound to different classes of C2-specific inhibitory antibodies. Each X-ray crystal structure was superimposed with the C2 domain structure and residues involved in the different epitopes are shown by stick representation (red: fVIII C2 domain; green: G99 mAb, a non-classical BC epitope; blue/cyan: 3E6 mAb, a classical A epitope; yellow: BO2C11 mAb, a classical AB epitope). (B) Proposed PS membrane binding models for the fVIII C2 domain (left: old PS binding model, including the non-classical epitope with residues K2227 and V2223; right: new PS binding model, including both 3E6 and BO2C11 classical antibody epitopes, which centers at residue R2320).

The A2 and C2 domains of fVIII harbor major immunogenic regions that are recognized by inhibitory antibodies [24–27]. Detailed epitope mapping of both domains further defines their respective epitope regions, and an antibody competition analysis demonstrates that the C2 domain possesses a complex, continuous spectrum of epitopes (types A, AB, B, BC and C), which serve to inhibit the cofactor function of fVIII through discrete mechanisms [25, 26]. From this study, “classical” inhibitory antibodies block fVIII cofactor function by inhibiting the ability of fVIII to bind negatively-charged, activated platelet surfaces, and these epitopes consist of the A, AB and B types [26]. It has been previously observed that the classical inhibitory antibodies either (A) block the ability of fVIIIa to bind activated platelet surfaces, thereby inhibiting the binding of fVIIIa to fIXa to form the intrinsic ‘tenase’ procoagulant complex, or (B) inhibit the binding of fVIII to VWF, which leads to rapid degradation and clearance of fVIII from circulation [26, 28–30]. By contrast, “non-classical” inhibitory antibodies prevent the proteolytic activation of fVIII by thrombin or fXa, thus inhibiting its release from VWF, and these consist of type BC and C epitopes [26, 31, 32]. Previous studies have shown that non-classical antibodies commonly develop in hemophilia A patients with inhibitor, and these antibodies have been characterized as being pathogenic [31, 32]. Detailed structural analyses of the fVIII C2 domain, both free and in complex with antibodies, have elucidated multiple anti-C2 epitopes at atomic resolution as well as defined a model for membrane association (Fig. 1) [13, 14, 33, 34]. Multiple studies implicate two solvent-exposed hydrophobic loops that protrude from the beta sandwich core of the fVIII C2 domain associate with the anhydrous interior of phospholipid membrane surface (L2251/L2252 and M2199/F2200) [11, 13, 14]. Complementary to this interaction is a ring of positively charged, basic residues adjacent to the hydrophobic loops, which are proposed to interact with the negative charge of phosphatidylserine (PS) on the surface of activated platelets [13, 14, 35]. The X-ray crystal structure of the C2 domain in complex with a classical inhibitory antibody, BO2C11, supports the model for membrane association, in which the hydrophobic loops of the C2 domain are completely sequestered by the CDR loops of the BO2C11 variable domains (Fig. 1A) [14]. Furthermore, the anti-C2 domain immunological response has been observed to accommodate both classical and non-classical antibodies simultaneously, with distinct, non-overlapping epitopes (Fig. 1A) [26]. Simultaneous binding of classical (3E6) and non-classical (G99) inhibitory antibodies has recently been described in structural detail, further supporting this observation and refining a working model of membrane association (Fig. 1B) [33, 34].

Treatments for hemophilia A patients with inhibitor and acquired hemophilia patients have been fairly limited, and have consisted of fVIII bypass agents [36], immunosuppressive therapy [37], and the infusion of highly purified porcine fVIII (Hyate:C) [38]. Porcine factor VIII has been used historically for treating hemophilia A patients with inhibitors due to its low cross-reactivity with anti-human fVIII antibodies, which results in a higher level of activity in comparison with human fVIII [39–41]. Hyate:C, or plasma-derived porcine fVIII, ceased from being administered to hemophilia A patients in 2005 due to concerns over porcine viral contamination [42], but a recombinant version of porcine fVIII has recently been approved for the treatment of hemophilia A patients with inhibitor as well as acquired hemophilia patients [43, 44].

Given the wealth of clinical data regarding porcine fVIII [38], as well as the observation of low cross-reactivity with human fVIII inhibitory antibodies [40, 41], it is informative to assess the structure and function of porcine fVIII. In this study, we have determined the X-ray crystal structure of the porcine fVIII C2 domain to 1.7 Å resolution and subsequently assessed its ability to bind PS-containing surfaces as well as classical type A (3E6) and non-classical type BC (G99) anti-human fVIII C2 domain antibodies [26]. The structure and complementary binding data provide further structural reasoning for the evasion of antibody cross-reactivity and supports a recently described working model for phospholipid membrane association (Fig. 1B) [33].

## Materials and Methods

### Cloning, Expression and Purification of Proteins

A codon-optimized gene encoding the porcine fVIII C2 domain (Leu 2171—Tyr 2332) was synthesized with in-frame *Bam*HI and *Xho*I restriction sites at the N- and C-termini, respectively (Genscript, Piscataway, NJ). The porcine C2 gene was excised from pUC57 cloning vector with *Bam*HI and *Xho*I and ligated into a pET27 derivative (pSV281) that contained a TEV protease-cleavable hexahistidine (His)_6_ N-terminal affinity tag. The expression construct was transformed into BL21(DE3) chemically competent cells for overexpression. Transformed cells were grown at 37°C to an OD_600_ of 0.7–0.8, and the temperature was subsequently lowered to 15°C. Protein overexpression was induced with 500 μM isopropyl β-D-thiogalactopyranoside (IPTG) for 16–20 hours. Following overexpression, the cells were centrifuged at 8,000 RPM for 10 minutes at 4°C (FIBERLite F10–6x500y rotor, Piramoon, Waltham, MA). The resulting pellet was sonicated for 60 seconds at 4°C with 0.5 mg/mL hen egg white lysozyme and 2 mM phenylmethanesulfonyl fluoride (PMSF) in Lysis Buffer [300 mM NaCl, 20 mM TrisHCl (pH 7.0), 10 mM imidazole, 2.5% (v/v) glycerol, 0.01% Triton X-100] with a ½” titanium horn attached to a Branson Sonifier 450 (Danbury, CT). The cell lysate was clarified by centrifugation at 16,000 RPM for 30 minutes at 4°C (FIBERLite F21–8x50y rotor, Piramoon), and the supernatant was sequentially filtered through a 5 and 0.45 micron filter, respectively. The filtered supernatant was allowed to incubate with TALON resin (Clontech, Mountain View, CA) for 60 minutes at 4°C with rocking. The TALON resin was allowed to settle and was washed with ten column volumes of Wash Buffer I [300 mM NaCl, 20 mM TrisHCl (pH 7.0), 10 mM imidazole, 2.5% (v/v) glycerol] and ten column volumes of Wash Buffer II [150 mM NaCl, 20 mM TrisHCl (pH 7.0), 10 mM imidazole, 2.5% (v/v) glycerol]. The porcine C2 domain was eluted from the TALON column with five column volumes of Elution Buffer [150 mM NaCl, 20 mM TrisHCl (pH 7.0), 150 mM imidazole, 10% (v/v) glycerol] and then dialyzed against High Salt Buffer [300 mM NaCl, 20 mM TrisHCl (pH 7.0), 10% (v/v) glycerol] overnight. Affinity-purified porcine C2 domain was further purified by size exclusion chromatography with a Superdex 75 column (GE Healthcare) at 1 mL/min in High Salt Buffer, and the purified fractions were buffer exchanged into Storage Buffer [300 mM NaCl, 20 mM TrisHCl (pH 7.0), 10% (v/v) glycerol], concentrated to 2 mg/mL, flash frozen in liquid nitrogen and stored at -80°C for future crystal trials. The human fVIII C2 domain and the 3E6 (classical) and G99 (non-classical) antibodies were expressed and purified as previously described [26, 33, 34].

### Crystallization and Structure Determination

Crystals suitable for X-ray crystallographic structure determination were grown by hanging drop vapor diffusion in 0.1 M CHES (pH 10.4), 0.1 M magnesium acetate, and 10% (v/v) ethanol in a 2:1 ratio of crystallization buffer and 2 mg/mL porcine C2 domain in Storage Buffer. Growth of large crystals occurred overnight at 4°C in the presence of 220 μL Al’s oil and were cryoprotected by a 1:1 addition of 0.1 M CHES (pH 10.4), 0.1 M magnesium acetate, and 30% (v/v) glycerol, where they were immediately flash frozen in liquid nitrogen. X-ray diffraction data were collected to 1.7 Å resolution on a Rigaku Micromax-007HF rotating anode with Confocal Varimax Optics Systems and an RAXIS 4++ detector at the Fred Hutchinson Cancer Research Center (FHCRC, Seattle, WA). Data collection, indexing and scaling of diffraction data were performed with CrystalClear and HKL2000, respectively [45]. Phases were determined by molecular replacement with the 1.5 Å X-ray crystal structure of the isolated human fVIII C2 domain as a search model using PHASER (pdb: 1D7P) [46]. Model building and refinement were completed with COOT and PHENIX, respectively [46, 47]. Validation of the completely refined model was performed with Molprobity and electrostatic calculations were performed with APBS [48, 49]. All structure figures and structural alignments were generated with PyMol.

### Enzyme-Linked Immunosorbent Assays (ELISAs)

All ELISA experiments were performed with Nunc Maxisorp 96-well ELISA plates (Fisher Scientific). To measure the binding of fVIII C2 domain proteins to membrane surfaces, 100 μL of 10 μg/mL 1,2-dioleoyl-*sn-*glycero-3-phospho-L-serine, 1,2-dioleoyl-*sn-*glycero-3-phosphoethanolamine, and 1,2-dioleoyl-*sn-*glycero-3-phosphocholine in a 20:20:60 ratio (DOPS/DOPE/DOPC, Avanti Polar Lipids, Inc.) dissolved in methanol was dried overnight in ELISA plates at room temperature [50, 51]. The DOPS/PE/PC-coated plates were subsequently blocked with 3% (w/v) bovine serum albumin in Tris-buffered saline (BSA-TBS) at 37°C for 60 minutes. Next, 100 μL of fVIII C2 domain (His)_6_ constructs serially diluted in 1% (w/v) BSA-TBS was added to the ELISA plate and incubated at 37°C for 90 minutes with shaking. The ELISA plate was washed three times with TBS (25 mM TrisHCl (pH 7.4), 150 mM NaCl, 2 mM KCl). For detection, HisDetector Ni-NTA conjugated to alkaline phosphatase (KPL, Gaithersburg, MD) was added at 1:1,500 dilution in 1% (w/v) BSA-TBS. Finally, colorimetric detection was achieved by the addition of para-nitrophenylphosphate (*p*NPP, MP Biomedicals, Santa Ana, CA), and absorbance measurements were collected at 405 nm with a Biotek Epoch microplate reader. Measurement of fVIII C2 domain constructs binding to the classical (3E6) and non-classical (G99) antibodies was performed as previously described [34]. Following ELISA data collection, binding curves were normalized and approximate equilibrium binding affinities were analyzed with GraphPad Prism as previously described [34].

### FVIII inhibitor assay

FVIII inhibitor titers were measured using the Bethesda assay [52] using previously described modifications [40]. Either BDD human fVIII or BDD porcine fVIII spiked into fVIII deficient plasma at 1 U/ml was used as the source of fVIII activity. One Bethesda unit (BU) per mL is defined as the dilution of inhibitor that produces 50% inhibition of fVIII activity. Inhibition curves were fitted by nonlinear least-squares analysis using the 4-parameter logistic equation to estimate the concentration of MAb producing 50% inhibition.

## Results

### The X-ray Crystal Structure of the Porcine Factor VIII C2 Domain

**Fig 2:**
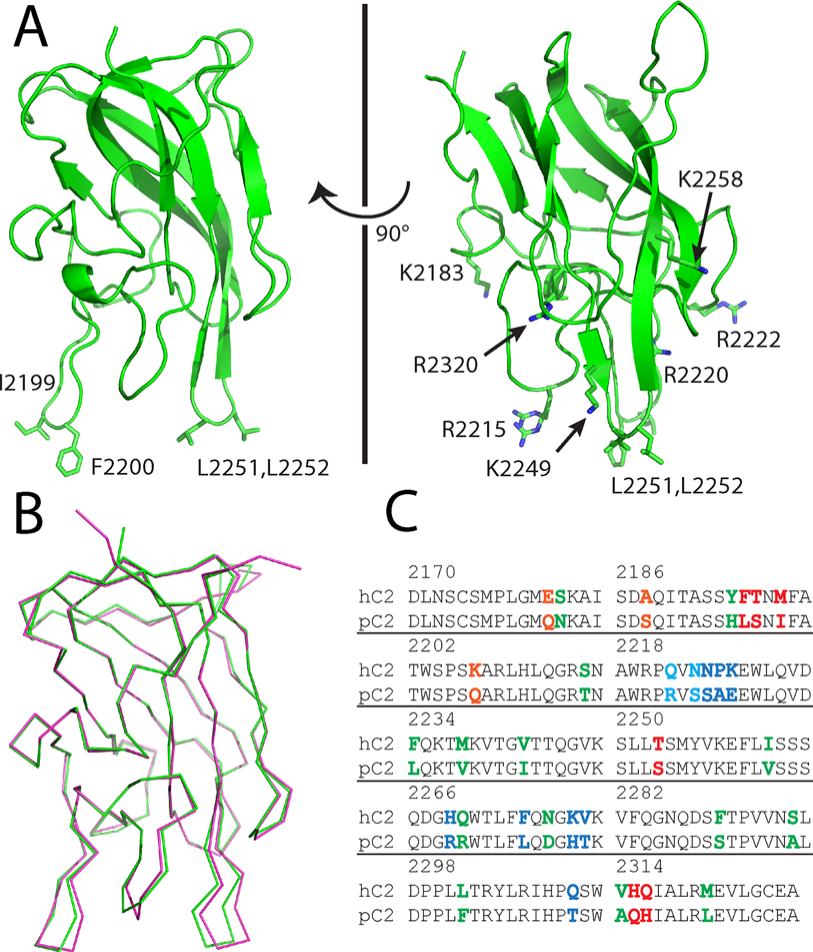
X-ray crystal structure of the porcine factor VIII C2 domain. (A) Ribbon diagram presentation of the 1.7 Å X-ray crystal structure. Displayed residues are solvent-exposed hydrophobic and basic residues proposed to interact with platelet surfaces. (B) Superposition of the human (pdb#: 1D7P, magenta) and porcine (pdb#: 4MO3, green) factor VIII C2 domain X-ray crystal structures. (3) Sequence alignment of human and porcine factor VIII C2 domains. Highlighted residues represent sequence differences (orange: 3E6 mAb binding region, blue: G99 mAb binding region, red: BO2C11 mAb binding region, cyan: G99 and BO2C11 binding region).

The porcine fVIII C2 domain, consisting of residues 2171–2332, was expressed, purified and crystallized as described in the Experimental Procedures above. The X-ray crystal structure was determined to a resolution of 1.7 Å (pdb code#: 4MO3) and refined to an R-work and R-free of 17.5% and 20.9%, respectively (Table 1). The electron density maps that were calculated from the molecular replacement solution allowed for unambiguous modeling of the majority of the fVIII C2 domain structure, with the exception of the N-terminal (His)_6_ affinity tag, residues 2171–2172 and residues 2329–2332. The structure of the porcine fVIII C2 domain adopts a discoidin-like β-sandwich fold with two β-hairpin turns that harbor solvent-exposed hydrophobic residues, as expected from the X-ray crystal structure of the human fVIII C2 domain (Fig. 2A) [13]. Structural alignment of the porcine and human fVIII C2 domain structures results in a root-mean-square deviation (RMSD) for the C-alpha atoms of 0.3 Å (Fig. 2B), which is consistent with the degree of sequence identity (80%) between the human and porcine homologs (Fig. 2C). The largest deviations between the two structures reside in the two hydrophobic β-hairpin turns as well as the adjacent Q2213—T2216 loop (Fig. 2B).

**Table 1:** X-ray data collection and model refinement statistics.

**X-ray data statistics**	
Wavelength (Å)	1.54
Resolution range (Å)	31.9–1.70 (1.79–1.70)
Space Group	I222
Unit Cell (Å)	a = 49.07, b = 68.94, c = 105.96
Total Reflections	139,818 (19,443)
Unique Reflections	19,602 (2741)
Redundancy	7.1 (7.1)
Completeness (%)	97.8 (95.6)
I/σ(I)	19.8 (8.9)
Rmerge	0.06 (0.17)
**Model Refinement Statistics**	
Rfactor (%)	17.52
Rfree (%)	20.93
Number of atoms (protein, water)	1263, 179
RMS bonds (Å)	0.006
RMS angles (°)	1.22
Ramachandran (%)	
Favorable	95
Allowed	5
Disallowed	0

**Fig 3:**
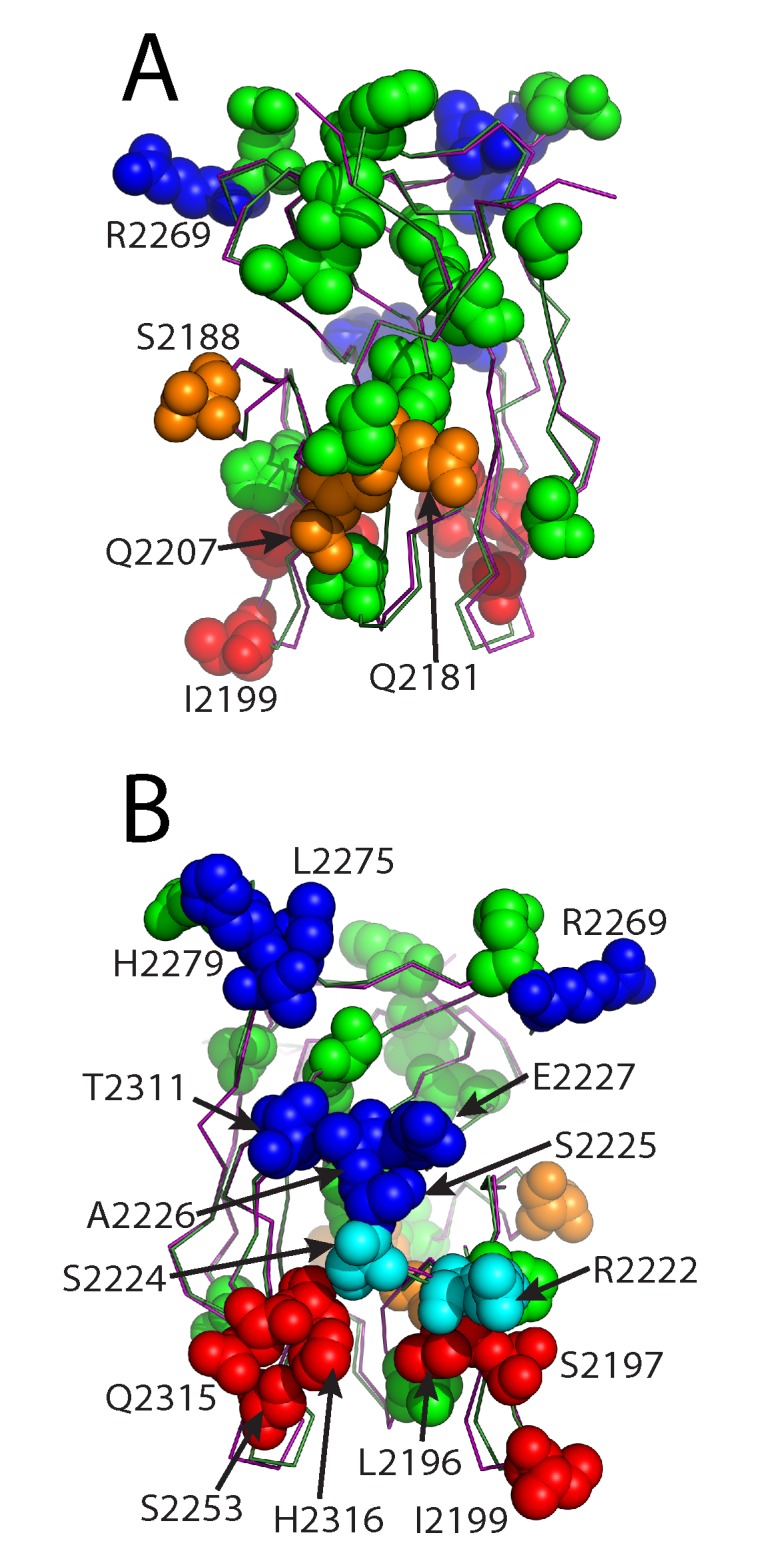
Structural representation of sequence differences proximal to inhibitory antibody epitopes. (A) The 3E6 mAb epitope face. (B) The G99 mAb epitope face. Proximal residues are defined as <5 Å C2 domain/mAb intermolecular distances (orange: 3E6 mAb binding region, blue: G99 mAb binding region, red: BO2C11 mAb binding region, cyan: G99 and BO2C11 binding region, green: >5 Å from all mAbs)

The high resolution X-ray crystal structure of the porcine fVIII C2 domain, along with a comparison of sequence differences with the human fVIII C2 domain and recent X-ray crystallographic evidence of inhibitory antibody epitopes, allows for an atomic description of the low cross-reactivity that is possessed by porcine factor VIII (Fig. 3) [33]. Strikingly, there is a dearth of sequence differences in the region recognized by type A classical inhibitory antibodies, with only 3 sequence differences being within 4–5 Å of the 3E6 classical (type A) antibody binding site (Figs. 2C, 3A). By contrast, the region of the fVIII C2 domain structure that harbors type BC non-classical epitopes displays 10 differences between the human and porcine sequences within 5 Å of the G99 binding site, and six of these make direct (<3.5 Å) hydrogen bonding or hydrophobic interactions with the G99 antibody (Figs. 2C, 3B). Lastly, the BO2C11 inhibitory antibody, which is a type AB classical inhibitor from a hemophilia A patient with inhibitor, is within 5 Å of eight sequence differences, five of which are within 3.5 Å. In total, there are 33 sequence differences between the human and porcine fVIII C2 domains. Of these, 19 differences are proximal to the established classical and non-classical antibody epitopes, and 10 of these are on the non-classical face (Fig. 3).

### Comparative Binding of Human and Porcine fVIII C2 Domains to Inhibitory Antibodies

**Fig 4:**
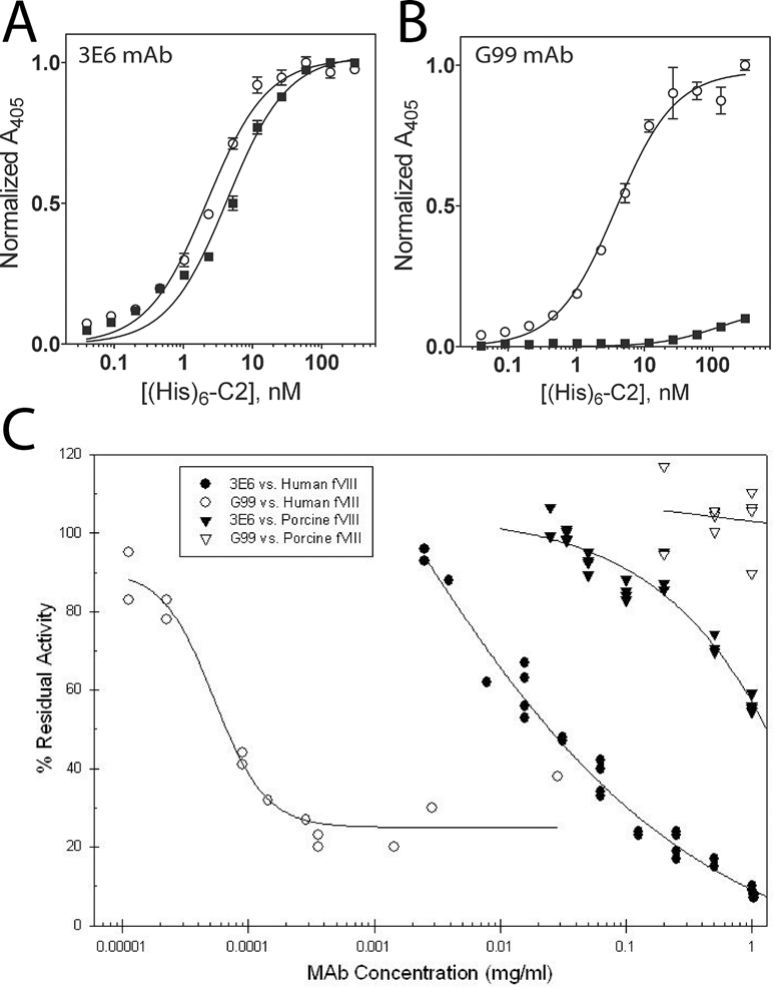
ELISA of human and porcine C2/mAb interactions. Binding of human (open circles) and porcine (closed squares) His_6_-C2 domain to immobilized (A) 3E6 mAb and (B) G99 mAb. Bound His_6_-C2 was detected with Ni-NTA-alkaline phosphatase. (C) Bethesda assay for 3E6 and G99 with human or porcine BDD fVIII.

To further understand the cross-reactive potentiality of porcine fVIII, both human and porcine fVIII C2 domains were analyzed for inhibitory antibody binding by ELISA. Following the immobilization of 3E6 and G99 IgG to ELISA plates, both human and porcine C2 domains were serially diluted and subsequently incubated in the ELISA plates, as previously described [34]. The apparent equilibrium dissociation constants (K_D_) were calculated from each binding curve (Fig. 4). The classical 3E6 antibody binds both the human and porcine C2 domains with similar affinities, with approximate K_D_ values of 2.2 and 4.3 nM, respectively (Fig. 4A). By contrast, a similar ELISA experiment performed with the non-classical G99 antibody indicates that the human C2 domain has an apparent K_D_ value of 7.0 nM, while the porcine C2 domain does not bind under the conditions tested (Fig. 4B). This decrease in binding affinity was expected based on both sequence differences between human and porcine C2 domains and the atomic structure of the C2/G99 interface. Previous structural data demonstrate that the central residue of the G99 epitope is K2227 in the human C2 domain structure, which is a glutamic acid in the porcine C2 domain, thus altering the overall charge of the G99 epitope. There is no inhibition of porcine fVIII in the Bethesda assay by G99 consistent with the lack of binding (Fig. 4C). However, although the K_D_ was only slightly higher to porcine C2 for 3E6, the inhibitory activity in the Bethesda assay was significantly less with inhibitory titers of 41 BU/mg IgG to human BDD fVIII and 0.8 BU/mg IgG to porcine fVIII. 3E6 also has a significantly higher IC_50_ for porcine fVIII binding to VWF than human fVIII binding to VWF (data not shown).

### Electrostatic Surface Potential Differences Between Human and Porcine C2 Domains

**Fig 5:**
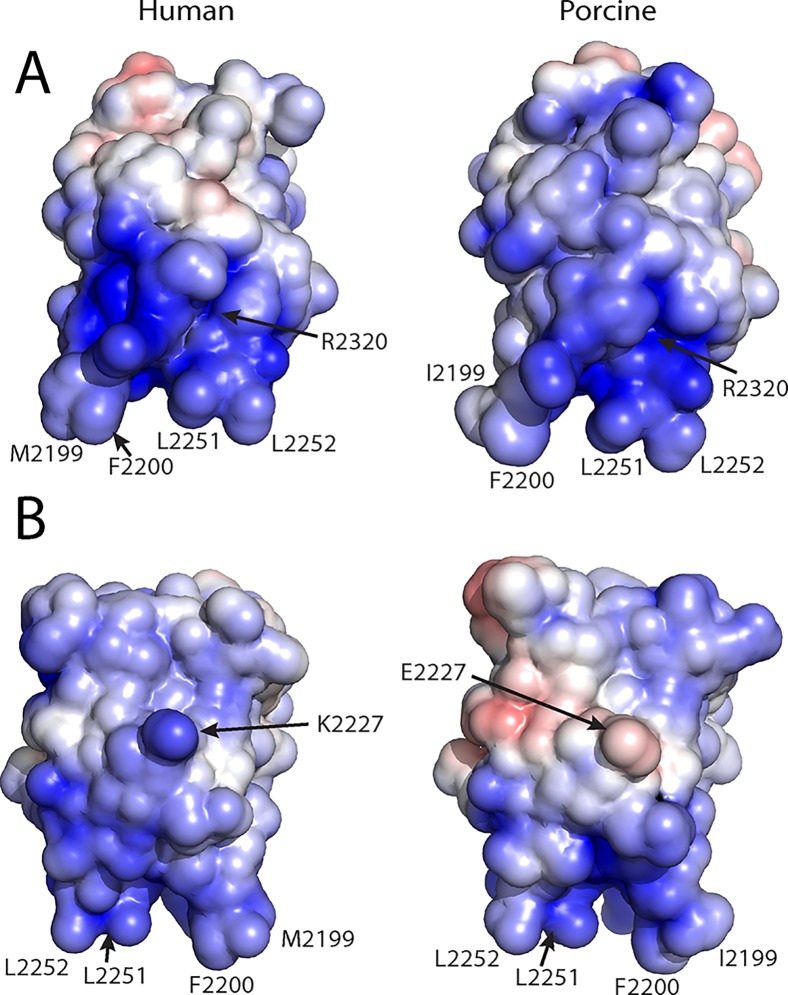
Electrostatic surface potentials for the human and porcine factor VIII C2 domain structures. (A) The 3E6 mAb epitope face. (B) The G99 mAb epitope face. The surface potential calculations were performed with APBS with surface potential values of ±5 kT/e, and the surface were generated with PyMol (blue: positive charge, red: negative charge).

High resolution X-ray crystal structures of both the human and porcine fVIII C2 domains allow for comparisons between the electrostatic surface potentials of each protein, which may provide further evidence for the mechanism by which fVIII associates with negatively charged membrane surfaces (Fig. 5). Upon detailed inspection of surface potentials for each protein, it is evident that the highest positive charge density for both the human and porcine variants resides on the 3E6 face of the C2 domain structure (Fig. 5A). In contrast to the classical 3E6 epitope electrostatics, the G99 face is markedly different between the human and porcine structures. Specifically, the presence of E2227 in the porcine structure possesses the opposite charge of the K2227 that is present in the human structure, thus having a dramatic effect on the electrostatic surface potential of the G99 epitope (Fig. 5B).

**Fig 6:**
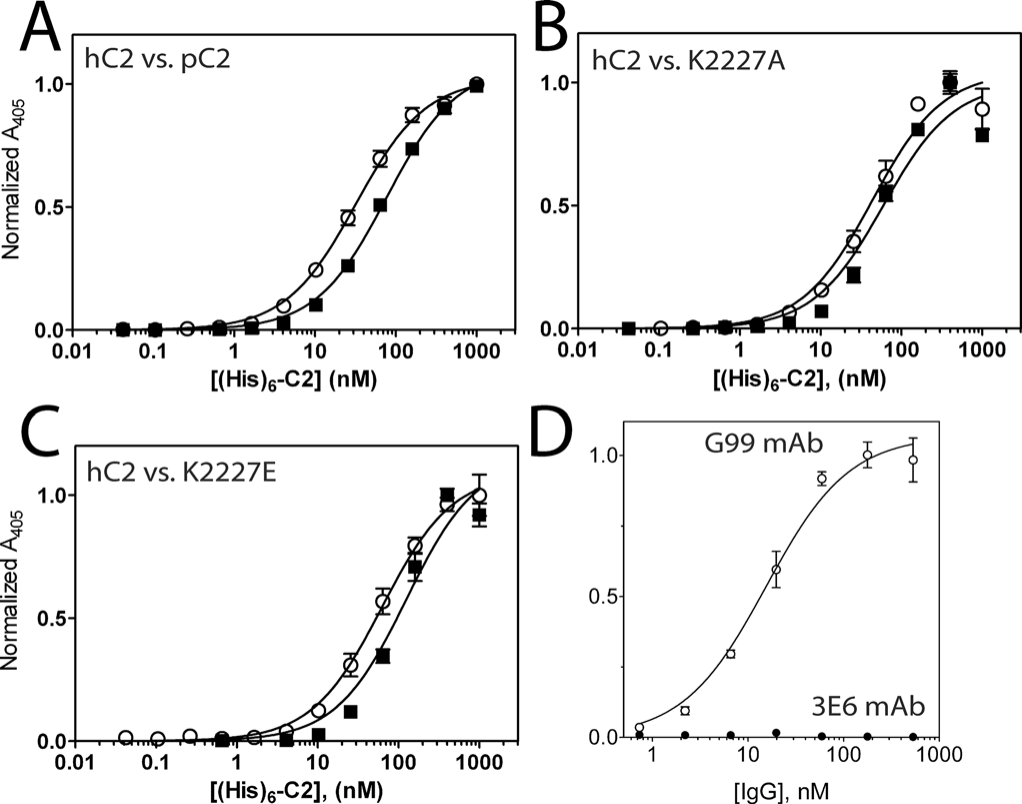
PS membrane and PS-bound fVIII C2 ELISA results for factor VIII C2 domain variants and inhibitory antibodies. (A) Comparison of human (open circles) and porcine (closed squares) factor VIII C2 domains binding to PS membrane surfaces. (B) Comparison of human factor VIII C2 domain (open circles) with the human K2227A C2 domain mutant (closed squares). (C) Comparison of human factor VIII C2 domain (open circles) with the human K2227E C2 domain mutant (closed squares). (D) Binding of G99 (open circles) and 3E6 (closed circles) to PS-bound fVIII C2 domain. Bound His_6_-C2 was detected with Ni-NTA-alkaline phosphatase, and bound IgG was detected with a AP-conjugated goat anti-mouse mAb.

Previous biochemical data have led to the hypothesis that K2227 forms a direct contact with the surface of negatively charged membrane surfaces, making it a strong determinant in platelet binding of fVIIIa (Fig. 1B) [11]. To further study the importance of K2227 in membrane binding, we performed a PS-binding ELISA, as previously employed to characterize the binding of C2 domain mutants to PS surfaces in other studies (Fig. 6) [51]. When comparing the PS binding behavior with human and porcine C2 domain titrations, the data presented herein illustrate comparable binding for the two proteins (Fig. 6A). The calculated K_D_ values for human and porcine C2 domains based on PS-binding ELISA curves were approximately 45 nM and 75 nM, respectively. To further inspect the importance of K2227, the K2227A and K2227E mutants of the human C2 domain were generated and subjected to the PS-binding ELISA, which resulted in comparable or modestly lower affinities as well, with approximate K_D_ values of 60 nM and 120 nM, respectively (Figs. 6 B,C). To assess which region of the fVIII C2 domain is solvent accessible in its membrane bound state, an additional ELISA was performed, where the fVIII C2 domain was initially immobilized on the PS-containing surfaces and subsequently incubated with both the 3E6 and G99 antibodies. The G99 IgG bound to the membrane-associated C2 domain with an apparent KD value of 15.5 nM while no binding was detected for the 3E6 IgG in this assay (Fig. 6D). Taken together, these data support a refined model of membrane association that tilts the fVIII C2 domain towards the 3E6 epitope, poising the G99 epitope to be facing outwards away from the PS membrane surface, as recently described in another study [33].

## Discussion

The C2 domain of fVIII is highly immunogenic and is a major recognition site for inhibitory antibodies, both associated with hemophilia A treatment as well as acquired hemophilia. While treatment options are limited, an established hemostatic agent is porcine fVIII due to its low cross-reactivity with anti-human fVIII antibodies [40, 41]. This study presents a high resolution X-ray crystal structure of the porcine fVIII C2 domain and further examination of its binding properties to inhibitory antibodies and negatively charged phospholipid surfaces for comparison with the human fVIII C2 domain.

The X-ray crystal structure of the porcine fVIII C2 domain is highly homologous to previously determined high resolution structures of the human fVIII C2 domain (Fig. 2B), as expected given the 80% sequence identity between the two proteins (Fig. 2C). Notably, the largest structural fluctuations between the human and porcine C2 structures occur at the two beta hairpin loops (2197–2202 and 2249–2254) as well as the adjacent Gln2213—Thr2216 loop, all of which are proximal to the proposed membrane binding region of the C2 domain and have been shown to adopt different conformations among various X-ray crystal structures of the human C2 domain previously (Fig. 2B) [14, 53].

The low cross-reactivity of porcine fVIII is presumed to be due to sequence differences between the human and porcine fVIII proteins. Mapping sequence differences onto the X-ray crystal structures and comparing these to antibody epitopes from different classes (classical and non-classical) is informative for the purpose of understanding the molecular basis of low cross-reactivity by porcine fVIII (Fig. 3). As described above, the classical type A epitope has a small number of sequence differences (3) in contrast to the non-classical type BC epitope (10 sequence differences). The mapping of phylogenetic sequence differences suggests that porcine fVIII will be most active in patients with non-classical inhibitory antibodies, whereas it may be less effective in the treatment of patients with classical type A epitope antibodies. Furthermore, the frequency of phylogenetic differences on the non-classical face of the protein, along with the observation that some of the sequence differences are not conservative, suggests that the face of the C2 domain that harbors the non-classical epitope is largely solvent accessible in circulation. By contrast, the face containing the classical epitope displays less phylogenetic differences, suggesting this region is responsible for binding other proteins in circulation, such as VWF, which is supported by previous findings [11, 29, 54].

The current working hypothesis for how fVIII binds negatively charged surfaces, primarily through the C1 and C2 domains, states that the solvent exposed, hydrophobic residues, which reside on the end of the beta hairpin loops, embed within the nonpolar nature of the membrane interior [55]. The favorable interaction with the negatively charged phospholipid headgroup of phosphatidylserine is also hypothesized as being accommodated by the ring of positively charged, basic residues that are poised directly above the hydrophobic beta hairpin turns. While this model is generally accepted, the orientation of fVIII, along with the C2 domain, is not well described. Previous mutagenesis data have suggested that the non-classical face of the fVIII C2 domain contributes to membrane binding [11], which is not consistent with recent structural findings [33, 34], nor previous membrane binding studies performed with full-length fVIII that show the 3E6 antibody inhibits fVIII binding to PS surfaces [26]. While the C2 domain has been the focus of membrane binding studies early, recent data illustrate that the C1 domain contributes to stability, membrane binding and cofactor function [12, 15, 56–58]. Membrane binding and fVIII cofactor activity studies have indicated that the C1 and C2 domain function cooperatively in membrane binding [57]. Moreover, removal of the C2 domain results in fVIII molecules that retain cofactor function [15], and replacing the C1 domain with a second C2 domain yields a protein with decreased stability and affinity for fIXa in the intrinsic tenase complex [58]. Thus, a caveat exists for the interpretation of membrane binding data collected with the isolated fVIII C2 domain.

In this study, the X-ray crystal structure of the porcine C2 domain, along with calculations of electrostatic surface potential and equilibrium binding data, support a model for PS membrane binding where the face containing the classical epitopes contributes directly (Figs. 1, 5). Specifically, previous studies indicated that K2227 of the human fVIII C2 domain is involved in PS membrane binding [11]. Contradictory data presented herein suggest an alternative model, as the porcine fVIII C2 domain binds PS surfaces with comparable apparent affinity despite the observation that amino acid residue 2227 is a glutamic acid in the porcine sequence (Fig. 6A). To further support this binding model, the K2227A and K2227E mutations were generated in the human C2 domain, which bound to PS membrane surfaces with either comparable or modestly lower affinities (Fig. 6B, C). In contrast to the non-classical face, both porcine and human C2 domains have high sequence conservation on the classical face, which is centered on Arg2320 (the identical position in the C1 domains of both human and porcine is also an Arg residue). To determine with epitope is solvent accessible for membrane-bound C2, the isolated C2 domain was bound to PS membrane surfaces and then titrated with both 3E6 and G99 IgG. The G99 IgG was observed to bind the immobilized C2 domain while the 3E6 IgG binding was not observed, which is consistent with previous results for full-length fVIII [26]. Based on these findings, in combination with previous structural data [33], the model of membrane binding centered at the classical face is further supported.

The development of an inhibitory immune response to fVIII remains a significant complication to both hemophilia A treatment as well as acquired hemophilia. To further understand the nature of this immune response, X-ray crystallographic structure determination combined with antibody binding studies provide a foothold from which new, more active and less immunogenic fVIII replacement therapeutics may be developed. Recent high-resolution epitope mapping of anti-C2 antibodies has provided a comprehensive study of epitope hotspots throughout the structure of the fVIII C2 domain [59]. Lastly, as a comprehensive structural understanding of fVIII cofactor function is progressing, we hypothesize that the inhibitory immune response for certain classes of anti-fVIII antibodies could be evaded through immune camouflage modifications, such as rationally designed polyethylene glycol (PEG) linkers or nucleic acid aptamers [60–62]. This approach, along with the development of fVIII sequence variants devoid of previously characterized antigenic hotspot residues, may result in new therapeutic agents for treating hemophilia patients with inhibitor.
